# Nonequilibrium Conditions Explain Spatial Variability in Genetic Structuring of Little Penguin (*Eudyptula minor*)

**DOI:** 10.1093/jhered/esv009

**Published:** 2015-04-01

**Authors:** Christopher P. Burridge, Amanda J. Peucker, Sureen K. Valautham, Craig A. Styan, Peter Dann

**Affiliations:** From the School of Biological Sciences, University of Tasmania, Private Bag 55, Hobart, Tasmania 7001, Australia (Burridge and Valautham); the School of Life and Environmental Sciences, Deakin University, Warrnambool, Victoria 3280, Australia (Peucker and Styan); the School of Energy and Resources, UCL Australia, Adelaide, South Australia 5000, Australia (Styan); and the Research Department, Phillip Island Nature Parks, Cowes, Victoria 3922, Australia (Dann).

**Keywords:** colonization, hybridization, isolation by distance, seabird, secondary contact, tension zone

## Abstract

Factors responsible for spatial structuring of population genetic variation are varied, and in many instances there may be no obvious explanations for genetic structuring observed, or those invoked may reflect spurious correlations. A study of little penguins (*Eudyptula minor*) in southeast Australia documented low spatial structuring of genetic variation with the exception of colonies at the western limit of sampling, and this distinction was attributed to an intervening oceanographic feature (Bonney Upwelling), differences in breeding phenology, or sea level change. Here, we conducted sampling across the entire Australian range, employing additional markers (12 microsatellites and mitochondrial DNA, 697 individuals, 17 colonies). The zone of elevated genetic structuring previously observed actually represents the eastern half of a genetic cline, within which structuring exists over much shorter spatial scales than elsewhere. Colonies separated by as little as 27 km in the zone are genetically distinguishable, while outside the zone, homogeneity cannot be rejected at scales of up to 1400 km. Given a lack of additional physical or environmental barriers to gene flow, the zone of elevated genetic structuring may reflect secondary contact of lineages (with or without selection against interbreeding), or recent colonization and expansion from this region. This study highlights the importance of sampling scale to reveal the cause of genetic structuring.

Knowledge of the factors influencing gene flow and genetic structuring among populations is essential for our understanding of evolutionary processes (Wright 1931; Slatkin 1987), and also for effective conservation of biodiversity through the documentation of demographically independent populations (Palsbøll et al. 2007; Durrant et al. 2014). Consequently, the ability to predict or generalize population genetic structuring is highly desirable (Bohonak 1999). For example, studies have tested for relationships between pelagic larval duration and population genetic structuring in marine fishes (Dawson et al. 2014), and between seed dispersal and speciation in terrestrial plants (Givnish 2010). However, in some instances there may be no obvious explanations for genetic structuring observed, or those apparent may actually represent spurious correlations.

In an effort to understand the factors most influential for genetic structuring among seabird colonies, Friesen et al. (2007a) conducted what is still the most recent review of relevant literature. Contemporary or historical barriers to gene flow of land or ice appeared significant (Steeves et al. 2005). Likewise, spatial segregation in nonbreeding or foraging distributions (Burg and Croxall 2001), and temporal segregation in breeding (difference in breeding phenology; Friesen et al. 2006; Friesen et al. 2007b), were also significant. In contrast, intercolony distance and dispersion pattern of colonies (1-dimensional arrangement along a linear coastline vs. a 2-dimensional oceanic arrangement) appear to weakly predict population genetic differentiation, although within several taxa correlations between genetic and geographic distance (isolation by distance) were observed (Friesen et al. 2007b). The generally high level of philopatry documented for seabirds may dictate population genetic structuring in the absence of other factors (Friesen et al. 2006). Conversely, levels of population genetic structuring lower than expected may reflect historical legacies of past gene flow (Austin et al. 1994). However, for some seabirds, the factors responsible for the observed population genetic structuring among colonies remain unresolved (Liebers and Helbig 2002; Overeem et al. 2008), and these deserve heightened attention given their greater potential to yield new insights into our understanding of this topic.

The little penguin, *Eudyptula minor* (Spheniscidae), is a flightless marine bird that breeds in colonies irregularly distributed throughout southern Australia, New Zealand, and associated islands (Marchant and Higgins 1990). Flipper-banding and radiotelemetry studies have shown that individuals travel much farther during the nonbreeding season than the breeding season (Dann 1992; Collins et al. 1999), and although natal philopatry is considered high (Stahel and Gales 1987; Marchant and Higgins 1990), there are direct observations of movement among colonies, and in some instances subsequent breeding (Reilly and Cullen 1982; Dann et al. 1995; Priddel et al. 2008). However, mark-recapture effort has been uneven across the species range (Sidhu et al. 2007), and in combination with the high incidence of postfledgling mortality (Sidhu et al. 2007), it is difficult to estimate what percentage of nonreturns might represent dispersal to nonnatal colonies. Genetic studies of other penguins have revealed both structuring at small spatial scales, and homogeneity at large scales (Ritchie et al. 2004; Jouventin et al. 2006), making predictions for other penguin taxa difficult.

Overeem et al. (2008) examined population genetic structuring in *E.minor* by scoring variation at mtDNA and 5 polymorphic microsatellite loci (from a pool of 17 screened) among 7 southeast Australian colonies (Figure 1). Only the 1 (microsatellites) or 2 (mtDNA) westernmost colonies were distinguished from the remainder, such that homogeneity was observed over large spatial scales, but heterogeneity was present at comparatively smaller scales in the western range of the study. The factors responsible for this spatial heterogeneity in genetic structuring were unclear. Observed differences in breeding phenology may have contributed to the genetic differences (Hendry and Day, 2005). Alternatively or additionally, cold water upwelling along the intervening Bonney Coast (Figure 1; Middleton and Bye 2007) may have provided an oceanographic barrier, similar to those apparently influential for diverse marine species including near-shore invertebrates (Thornhill et al. 2008) and seabirds (Gómez-Diaz et al. 2006), including other penguin species (Banks et al. 2006; Jouventin et al. 2006). The comparatively low spatial genetic structuring in the eastern study range could also reflect historical legacies of colony establishment in Bass Strait following post-Last Glacial Maximum (LGM) sea level rise (Lambeck et al. 2002).

**Figure 1.**
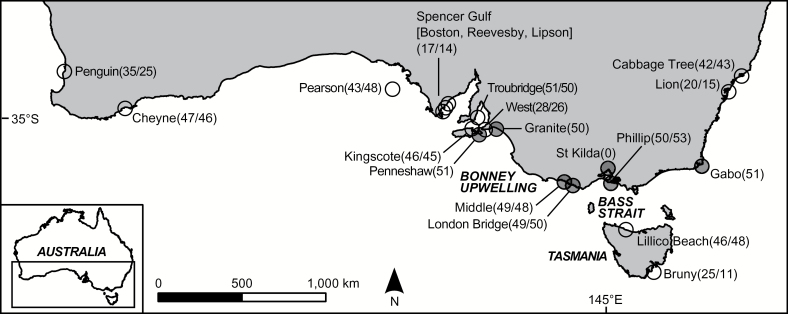
Sampled colonies of *E.minor*. Those analyzed by Overeem et al. (2008) are indicated by shaded circles. Numbers in parentheses denote individuals analyzed during this study for microsatellite/mtDNA variation (single number if sample sizes identical).

Here, we analyzed 11 additional colonies relative to Overeem et al. (2008), and also collected data from 7 additional microsatellite loci, to test these factors as explanations for the spatial heterogeneity in genetic structuring of *E.minor*. In total, this represented a dataset of 697 individuals across 17 colonies, genotyped for 12 microsatellite loci and mtDNA variation. By expanding the study range, we include other colonies that differ in breeding phenology, geographic separation, and the presence or absence of intervening oceanographic features. However, we document that the zone of elevated genetic structuring actually corresponds to the inflection point of a genetic cline. Therefore, the previous hypotheses for genetic structuring most likely reflect spurious correlations of genetic divergence with oceanographic features and phenological differences. Analysis was conducted to investigate whether the genetic cline is being maintained by selection, or represents nonequilibrium genetic structuring following recent colonization or secondary contact of lineages.

## Materials and Methods

A total of 17 Australian colonies were analyzed during this study (Figure 1). Eleven of these were visited from August 2004 to September 2006 to collect blood samples, while the remaining 6 were sampled by Overeem et al. (2008) prior to 2004. Approximately 50 individuals were sampled from the majority of colonies, however fewer were collected from some owing to collection difficulties and permit restrictions (Figure 1). Three colonies in Spencer Gulf were considered as a single colony given their proximity and sample sizes of less than 15 individuals (Lipson, Reevesby, and Boston Islands, less than 60 km apart). The Troubridge Island colony is also potentially young due to its location on a low sand island, and some analyses were repeated excluding it to examine potential influence on interpretations of genetic structuring. Animal capture, sample collection, and DNA extraction were undertaken as described in Peucker et al. (2009).

Variation in mitochondrial haplotype frequencies among populations was assessed using the polymerase chain reaction restriction fragment length polymorphism (PCR-RFLP) approach described in Overeem et al. (2008) based on the mitochondrial control region. While this approach revealed a smaller number of haplotypes than direct sequencing of the same mtDNA region (14 haplotypes vs. 42; Overeem et al. 2008; Peucker et al. 2009), their frequencies were suitable for robust comparisons among colonies (0.20–0.91), rather than comprising a large number of rare haplotypes.

A total of 12 microsatellite loci were employed for analysis. These comprise 4 of the 5 loci employed by Overeem et al. (2008) (locus *G2-2* discontinued owing to consistent deviation from Hardy–Weinberg expectations). An additional 8 loci were employed from Billing et al. (2007). All loci were multiplex PCR amplified using the QIAGEN Multiplex Kit as per manufacturer’s instructions in a reaction mix of 6.25 µL, containing 2 µL of DNA, and 0.2 µM of each primer. Thermal cycling followed the Multiplex Kit protocol, with annealing at 57 °C. Fragments were separated on an ABI 3130 genetic analyser (Applied Biosystems Inc.) and sized relative to the GS500 standard. Two “control” individuals were analyzed with each batch to ensure consistency of scoring.

Selective neutrality of mitochondrial haplotypes was assessed using the Ewens–Watterson–Slatkin test (using Slatkin’s exact *P* value) with Arlequin (Version 3.5.1.2; Schneider et al. 2000). Allele frequencies in each population were tested for deviations from Hardy–Weinberg and genotypic equilibrium using Genepop version 3.4 (Raymond and Rousset 1995). Tests of Hardy–Weinberg equilibrium were performed using an “Exact H-W test”, with complete enumeration of *P* values whenever there were less than 5 alleles (Louis and Dempster 1987), otherwise estimation of *P* values via 1000 Markov chain batches (Guo and Thompson 1992). Tests of genotypic equilibrium used the log-likelihood ratio statistic and 1000 Markov chain batches (Raymond and Rousset 1995). Benjamini–Yekutieli correction (Narum 2006) was applied for simultaneous tests. Tests for evidence of scoring errors due to stuttering, large allele dropout, and the presence of null alleles were conducted using Micro-Checker (Version 2.2.3, van Oosterhout et al. 2004). Average number of alleles, expected heterozygosity and *F*_IS_ were calculated using GenoDive 2.0b24 (Meirmans and Van Tienderen 2004), and haplotype diversity was calculated using Arlequin. Multilocus linkage disequilibrium for each population was calculated as ${\bar{r}}_{d}$ using Multilocus 1.3 (Agapow and Burt 2001).

Microsatellite allele and mitochondrial haplotype frequency homogeneity between colonies was examined using *G* tests in Genepop. Theta (*θ*), an estimate of genetic distance between populations (Weir and Cockerham 1984), was calculated across all populations and between all populations using FSTAT version 2.9.3 (Goudet 2001), on both mtDNA and microsatellite datasets. Mantel tests (10 000 randomizations) were employed for correlation between linearized *θ* (*θ* /1 − *θ*) and the shortest marine distance (within 20 km of the coast; Weavers 1992) between colonies using IBDWS (Jensen et al. 2005). Reduced major axis regression was employed to estimate the slope of each relationship, given the amount of error potentially associated with measurement of the independent variable (Sokal and Rohlf 1981; Hellberg 1996). Confidence intervals of the slope of relationships were obtained by bootstrapping over independent population pairs.

Bayesian model-based clustering of individuals was performed using STRUCTURE (Version 2.3.3, Pritchard et al. 2000), with 100000 Markov chain batches under the population admixture model with correlated allele frequencies, and potential values of *K* (number of population clusters) between 1 and 6. Selection of the optimum number of clusters followed the Δ*K* method of Evanno et al. (2005), based on 20 replicate analyses for each value of *K*, assessed using STRUCTURE HARVESTER (Earl and vonHoldt 2012).

Given observations of a sharp cline in STRUCTURE coancestry coefficients in the middle of the sampling distribution, both microsatellite and mitochondrial DNA variation were tested for coincidence of cline centers, and therefore whether there was evidence for selection against interbreeding among lineages (i.e., a tension zone; Barton and Hewitt 1985). Only colonies located along the coastline from Cheyne Island in the west to Gabo Island in the east were analyzed, providing a roughly single (coastline distance) dimension for analysis. In contrast to cline analyses based on biallelic loci (Gay et al. 2008; Kawakami et al. 2009; Taylor et al. 2012), the diversity and frequencies of alleles across the cline at most microsatellite loci precluded the unequivocal assignment of alleles to lineages. Therefore, cline fitting was performed on single locus coancestry coefficients obtained from STRUCTURE analysis (Miraldo et al. 2013), treating them as quantitative data. Similarly, mitochondrial DNA variation did not suggest the presence of 2 distinct clusters or clades of haplotypes that could be used directly in cline analysis (Miraldo et al. 2013; Smith et al. 2013). Haplotype data were subjected to multiple correspondence analysis using PAST (Hammer et al. 2001), and first axis scores for each haplotype (40.8% of total variation) were then employed as quantitative data.

A maximum likelihood approach to cline analysis (Szymura and Barton 1986) was used to fit a sigmoidal curve under a unimodal model of trait distribution, employing CFit7 (Gay et al. 2008). The coincidence of microsatellite and mtDNA clines was assessed by a likelihood ratio test, with clines either forced to have the same center, or allowed to have different centers.

In fulfillment of data archiving guidelines (Baker 2013), we have deposited the primary data underlying these analyses with Dryad.

## Results

Mitochondrial PCR-RFLP analysis of 674 individuals across 17 colonies produced 14 haplotypes. The number of haplotypes per colony ranged from 2 (at Troubridge and Bruny Island) to 10 (Granite Island) with a mean of 6.47 per colony (Table 1). Microsatellite analysis was conducted on 697 individuals across 17 colonies. The 12 loci exhibited between 1.33 and 7.11 alleles on average per colony, and mean heterozygosity (*H*_e_) of loci per colony ranged from 0.05 to 0.85 (Table 1).

**Table 1.** Genetic variation at mitochondrial and microsatellite loci, represented by number of alleles, gene diversity (heterozygosity or haplotype diversity), and estimates of *F*_ST_ (*θ*).

Locus	Number of alleles (colony averages)	Mean colony gene diversity: *H*_e_ or *h*	*θ*	*P*[*θ* = 0]
*mtDNA*	6.47	0.65	0.161	0.001
*AM13*	2.83	0.45	0.008	0.025
*B3-2*	4.66	0.74	0.018	0.001
*G3-11*	1.45	0.06	0.010	0.009
*Sh1Ca9*	1.97	0.22	0.036	0.001
*Emm1*	3.54	0.65	0.016	0.001
*Emm2*	7.10	0.74	0.013	0.001
*Emm3*	5.59	0.70	0.041	0.001
*Emm4*	1.33	0.05	0.037	0.001
*Emm5*	7.11	0.85	0.012	0.001
*Emm6*	2.63	0.40	0.010	0.001
*Emm7*	4.45	0.68	0.014	0.001
*Emm8*	1.70	0.11	0.051	0.001
all microsatellites	3.70	0.47	0.018	0.001

The Ewens–Watterson–Slatkin exact test only rejected selective neutrality of mtDNA variation for the Spencer Gulf composite sample, which may reflect the low sample size and pooling of data from proximate colonies. Genotype frequencies at each microsatellite locus were consistent with Hardy–Weinberg equilibrium (*P* > 0.05), and there was no evidence of scoring errors due to stuttering, large allele dropout, or null alleles at any of the loci as assessed by Microchecker. Independence of genotypes among microsatellite loci was rejected for only 9 tests following Benjami–Yekutieli correction—none of which involved the same comparison of loci among populations. When ignoring correction for simultaneous tests, no more than 5 out of 17 populations exhibited *P* < 0.05 for the same comparison of loci. Therefore, genotypes among loci were considered independent.

**Figure 2.**
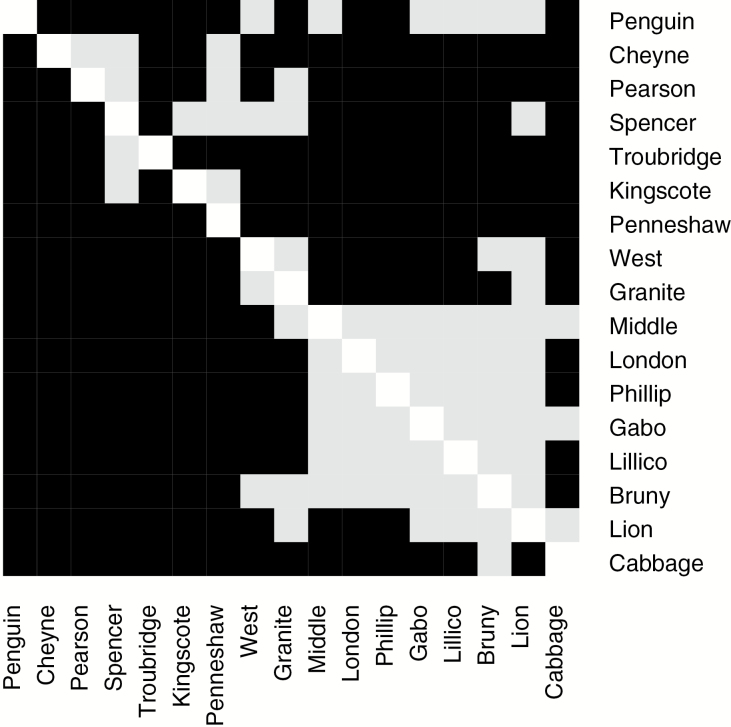
Exact test results from pairwise comparisons of allele frequencies among colonies (ordered from west to east). Microsatellite loci below the diagonal, mitochondrial DNA above the diagonal. Black squares indicate significant difference in allele frequencies following Benjamini–Yekutieli correction (Narum 2006), and light grey indicates nonsignificance. *P* values are provided, see Supplementary Table 1 online.

Pairwise estimates of *θ* (Table 2) and results from exact tests (Figure 2; see Supplementary Table 1 online) failed to reject genetic homogeneity of most colonies in southeast Australia, while homogeneity was rejected elsewhere, often over much smaller spatial scales. Samples collected between West Island and Lion Island, including the 2 Tasmanian colonies, were homogeneous for microsatellite allele frequencies in the majority of pairwise tests (Figure 2), with the exceptions that Lion, West, and Granite Islands could be distinguished from up to 5 colonies within this region. Colonies outside this region to the west, and also Cabbage Tree Island to the east, were individually genetically distinct from all other colonies with few exceptions, even when proximate (e.g., Penneshaw vs. Kingscote, 27 km apart by sea). The Spencer Gulf composite sample could not be distinguished from several colonies, both proximate and distant, but this may pertain to the small size of this pooled sample. Mitochondrial DNA variation exhibited greater divergence among colonies as quantified by *θ* than the microsatellite loci (Table 1), but did not distinguish as many colonies during pairwise comparisons as microsatellites, although those distinguished by mtDNA were usually distinguished by microsatellites (Figure 2; see Supplementary Table 1 online). The small Spencer Gulf sample was infrequently distinguished from other colonies based on mtDNA haplotype frequencies.

**Table 2:** Pairwise estimates of *F*_ST_ (*θ*) among colonies

	Penguin Island	Cheyne Island	Pearson Island	Spencer Gulf	Troubridge Island	Kingscote	Penneshaw	West Island	Granite Island	Middle Island	London Bridge	Phillip Island	Gabo Island	Lillico Beach	Bruny Island	Lion Island	Cabbage Tree Island
Penguin Island		0.360^a^	0.253^a^	0.291^a^	0.543^a^	0.300^a^	0.336^a^	0.200^a^	0.208^a^	0.009	0.116^a^	0.124^a^	0.037	0.034	0.002	−0.010	0.117
Cheyne Island	0.036^a^		0.034	0.046	0.247^a^	0.123^a^	0.042	0.114^a^	0.052^a^	0.261^a^	0.248^a^	0.156^a^	0.223^a^	0.273^a^	0.432^a^	0.263^a^	0.162^a^
Pearson Island	0.060^a^	0.021^a^		0.013	0.178^a^	0.062^a^	0.015	0.079^a^	0.024	0.173^a^	0.178^a^	0.119^a^	0.151^a^	0.185^a^	0.324^a^	0.183^a^	0.139^a^
Spencer Gulf	0.041	0.044^a^	0.034		0.256^a^	−0.017	0.008	0.054	0.022	0.173^a^	0.164^a^	0.099^a^	0.147^a^	0.181^a^	0.366^a^	0.159	0.133
Troubridge Island	0.065^a^	0.071^a^	0.043^a^	−0.008		0.206^a^	0.154	0.311^a^	0.179^a^	0.444^a^	0.404^a^	0.351^a^	0.426^a^	0.430^a^	0.599^a^	0.470^a^	0.442^a^
Kingscote	0.049^a^	0.050^a^	0.039^a^	−0.005	0.000		0.022	0.090^a^	0.056^a^	0.214^a^	0.194^a^	0.153^a^	0.200^a^	0.211^a^	0.341^a^	0.209^a^	0.219^a^
Penneshaw	0.050^a^	0.035^a^	0.012	0.014	0.033^a^	0.012		0.089^a^	0.036	0.245^a^	0.220^a^	0.159^a^	0.221^a^	0.247^a^	0.389^a^	0.252^a^	0.215^a^
West Island	0.029	0.021	0.021	0.021	0.043^a^	0.027	0.029^a^		0.016	0.093^a^	0.085^a^	0.027	0.068	0.090^a^	0.228^a^	0.085	0.096^a^
Granite Island	0.011	0.017	0.012	0.010	0.031^a^	0.023^a^	0.015	0.004		0.119^a^	0.103^a^	0.049^a^	0.099^a^	0.115^a^	0.256^a^	0.113^a^	0.101^a^
Middle Island	0.049^a^	0.020^a^	0.020^a^	0.046^a^	0.061^a^	0.047^a^	0.032^a^	0.016	0.009		0.044	0.043^a^	−0.012	−0.008	0.045	−0.027	0.048
London Bridge	0.048^a^	0.020^a^	0.028^a^	0.044^a^	0.057^a^	0.030^a^	0.034^a^	0.022	0.002	0.000		0.006	0.032	0.012	0.142	0.051	0.095^a^
Phillip Island	0.047^a^	0.026^a^	0.029^a^	0.023	0.054^a^	0.028^a^	0.018	0.016	0.000	0.011	−0.001		0.021	0.028	0.165	0.040	0.040
Gabo Island	0.039^a^	0.010	0.021	0.026	0.038^a^	0.016	0.032^a^	0.014	0.007	0.017	0.002	0.014		−0.005	0.076	−0.016	0.021
Lillico Beach	0.046^a^	0.025^a^	0.005	0.036	0.037^a^	0.034	0.022	0.012	0.006	0.013	0.024	0.019	0.006		0.051	−0.014	0.071
Bruny Island	0.026	0.019	0.022	−0.006	0.031	0.012	0.024	0.004	−0.013	0.000	0.000	−0.008	−0.011	0.002		0.041	0.197
Lion Island	0.035	−0.007	0.000	−0.011	0.043^a^	0.017	0.003	−0.008	−0.029	0.011	−0.002	−0.02	−0.009	−0.011	−0.011		0.042
Cabbage Tree Island	0.059^a^	0.045^a^	0.035	0.004	0.025^a^	0.040^a^	0.051^a^	0.028^a^	0.018	0.038^a^	0.041^a^	0.042^a^	0.025^a^	0.025^a^	0.006	0.002	

Microsatellite loci below the diagonal, mitochondrial DNA above the diagonal.

^a^Significant following Benjamini–Yekutieli correction.

**Figure 3.**
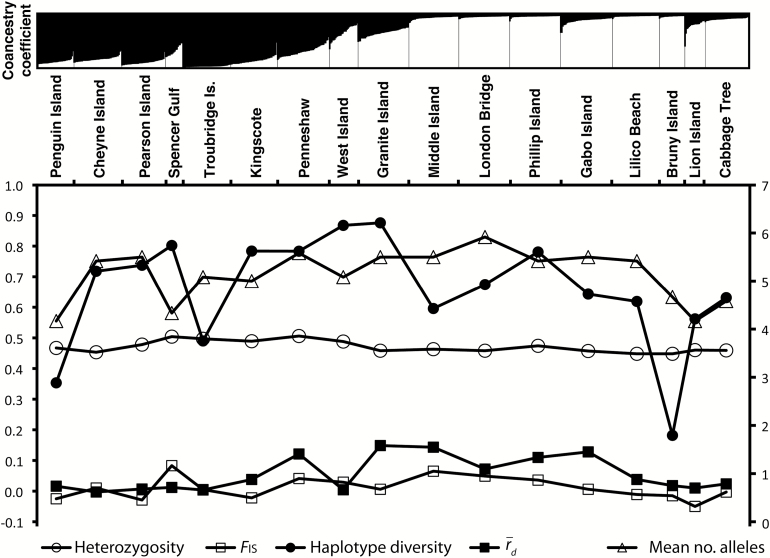
Top panel: estimated individual coancestry proportions from STRUCTURE analysis, assuming 2 clusters (*K* = 2). Each individual is represented by a vertical bar of height proportional to its estimated coancestry to one of the clusters. The source of individuals is demarcated along the bottom of the chart. Plots for *K* > 2 are provided in Supplementary Figure 2 online. Bottom panel: measures of genetic variation within populations, comprising mean expected heterozygosity, *F*_IS_, number of alleles across microsatellite loci, multilocus linkage disequilibrium (${\bar{r}}_{d}$), and haplotype diversity. All variables except number of alleles are scaled according to the *y* axis on the left.

The Δ*K* method suggested that *K* = 2 was the optimum number of clusters for the data based on STRUCTURE analysis (Figure 3; Δ*K* results and structuring inferred from *K* > 2 are provided, see Supplementary Figures 1 and 2 online), but there is no suggestion of a hard genetic break among samples; a continuous transition in coancestry coefficient occurs from a western group to an eastern group (shown in black and white, respectively, Figure 3), which is particularly pronounced when moving between Troubridge and Middle Island.

**Figure 4.**
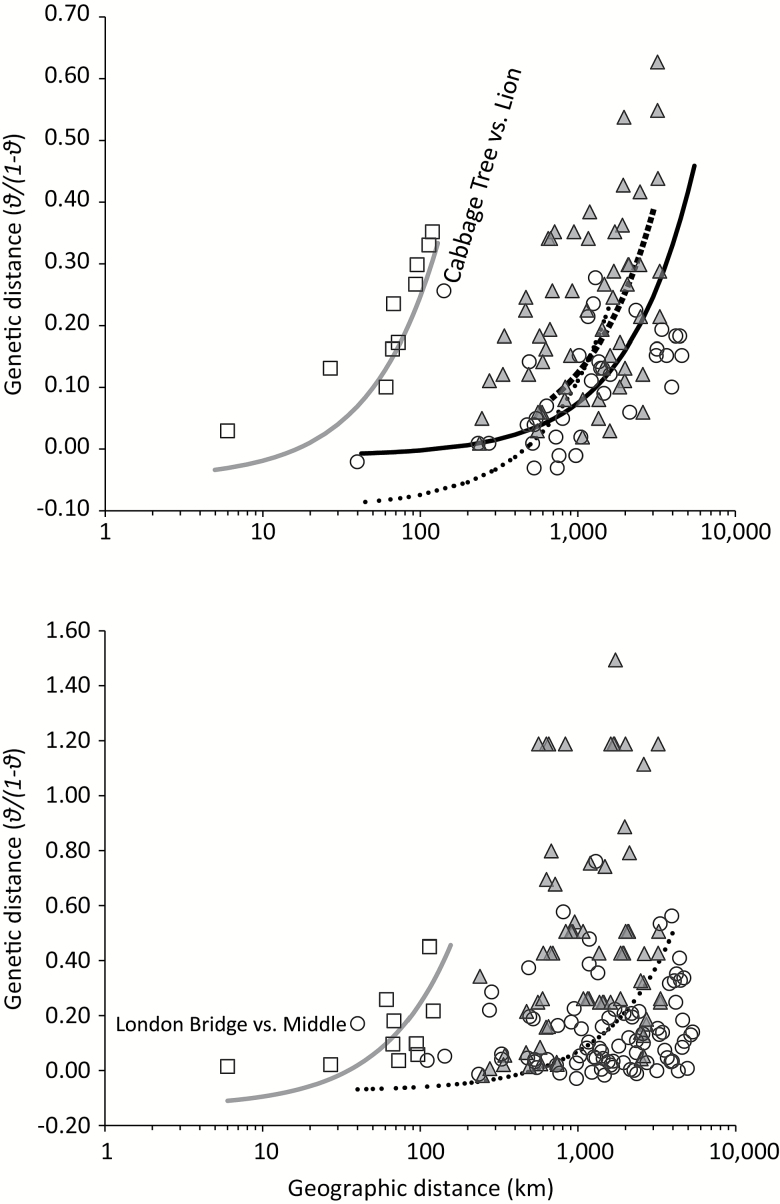
Isolation-by-distance plots for microsatellites (top) and mtDNA (bottom). Squares indicate comparisons of colonies located between Troubridge and Granite Island, circles indicate comparisons of colonies outside this zone, and triangles represent comparisons involving one colony from each. Where significant correlations were observed, grey and black lines indicate results from reduced major axis regression based on comparisons inside and outside the Troubridge–Granite zone, respectively. Dashed black lines indicate significant correlations based on comparisons east (dots) or west (squares) of the Troubridge–Granite zone.

A significant isolation-by-distance (IBD) relationship was evident throughout the entire study range for microsatellites (*Z* = 5003.31, *r* = 0.44, *P* = 0.02), but not for mtDNA (*Z* = 46121.46, *r* = 0.13, *P* = 0.15). We also tested whether the IBD relationship differed regionally, based on the spatially variable scales of genetic differences observed. Significant IBD relationships for microsatellites were obtained from analysis of colonies between Troubridge and Granite Island (*Z* = 18.31, *r* = 0.93, *P* = 0.01). A significant relationship was also observed based on all colonies from outside this region (*Z* = 2808.52, *r* = 0.67, *P* < 0.01; Figure 4), but the slope of this relationship (8.53×10^−6^, 95% CI −2.25×10^−5^ – 2.00×10^−5^) was weaker than that for the colonies between Troubridge and Granite Island (2.93×10^−4^, 95% CI 1.21×10^−4^–1.03×10^−3^) (Figure 4). Significant IBD was present among colonies east of Granite Island (*Z* = 232.73, *r* = 0.43, *P* = 0.03), and also west of Troubridge Island (*Z* = 315.68, *r* = 0.46, *P* = 0.04), and the slopes of these relationships (east of Granite, slope = 2.06×10^−5^, 95% CI −4.67×10^−5^–5.04×10^−5^, west of Troubridge slope = 1.30×10^−5^, 95% CI 2.02×10^−5^ – 6.29×10^−5^) were similar to that obtained from all colonies outside the Troubridge–Granite zone, and shallower in comparison to that for the Troubridge–Granite subset of colonies (Figure 4). Analyses were repeated for a Kingscote–Granite subset, without Troubridge Island, as it was genetically the most divergent of the populations in this region (Table 2) and could be influencing the IBD relationship. However, the results were similar (microsatellites: *Z* = 8.10, *r* = 0.95, *P* = 0.04, slope = 2.66×10^−4^, 95% CI 4.48×10^−4^ – 4.84×10^−4^)

A significant IBD relationship was observed for mtDNA within the Troubridge–Granite subset (*Z* = 103.79, *r* = 0.59, *P* = 0.04), but not for comparisons of all colonies outside this region (*Z* = 29687.30, *r* = 0.17, *P* = 0.14) (Figure 4). A significant mtDNA IBD relationship was present east of Granite Island (*Z* = 1420.40, *r* = 0.34, *P* = 0.05), but not west of Troubridge Island (*Z* = 5291.03, *r* = 0.25, *P* = 0.17). As for microsatellites, the slope of the relationship inside the Troubridge–Granite subset (3.80×10^−3^, 95% CI −2.70×10^−2^ – 2.18×10^−2^) was steeper than that outside (i.e., east of Granite, 1.45×10^−4^, 95% CI −2.93 x 10^−4^ – 3.14×10^−4^) (Figure 4). Exclusion of Troubridge from the Troubridge–Granite subset reduced the significance of the IBD relationship, perhaps owing to a smaller sample size (*Z* = 24.97, *r* = 0.73, *P* = 0.13).

**Figure 5.**
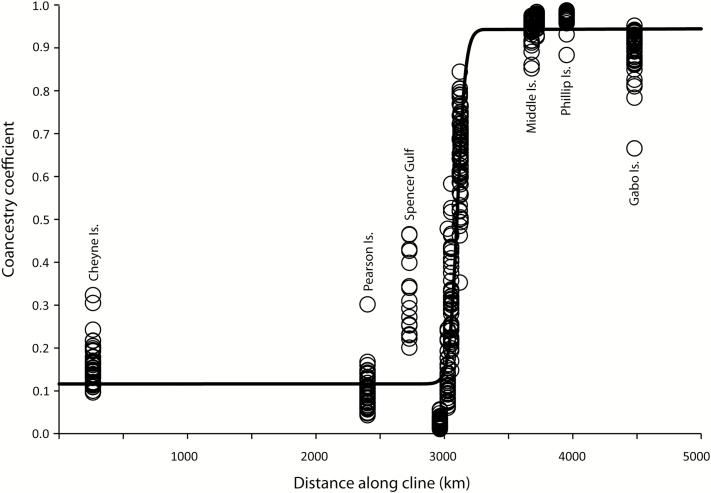
Cline in coancestry coefficients from STRUCTURE analysis of multilocus microsatellite variation, along a transect spanning southern Australian colonies of *E.minor* (from Cheyne Island to Gabo Island). Circles represent coancestry coefficients of individuals at colonies to 1 of 2 groups during STRUCTURE analysis. Line represents the maximum likelihood fit of a sigmoidal curve.

Analysis of multilocus microsatellite data revealed a cline centered 17 km west of West Island (Figure 5). The distribution of mtDNA variation was less obviously clinal (see Supplementary Figure 3 online). However, the estimated cline center was similar to that obtained from microsatellites (located 4 km east of Granite Island, 27 km east of that inferred for microsatellites). A likelihood ratio test revealed that analysis with both microsatellite and mtDNA clines constrained to have the same center (coincidence, 16 km west of West Island) was not significantly worse than that where clines were allowed to have different centers (χ^2^ = 0.034, d.f. = 1, *P* = 0.84). Analysis of microsatellite loci individually revealed a geographic spread of cline centers (spanning 1662 km; see Supplementary Figure 4 online). Clines could not be adequately fitted to variation at loci *Emm4*, *AM13*, and *G3-11*. Constraining clines fitted for individual microsatellite and mtDNA loci to have the same center (coincidence, 16 km west of West Island) was not significantly worse than that where clines were allowed to have different centers (χ^2^ = 0.384, d.f. = 9, *P* = 0.99).

## Discussion

The most striking observation from this study was the different geographic scales at which significant population genetic structuring was observed. Spatial structuring of genetic variation was weak among colonies in southeast Australia, matching previous observations of Overeem et al. (2008). In contrast, to the west of this zone significant genetic heterogeneity among colonies existed at much finer spatial scales (as little as 27 km), particularly in the region between Troubridge and Granite Island. No obvious barriers to gene flow exist in this region, and instead there appears to be a linear relationship between genetic and geographic distance, which is steeper than that observed among colonies outside this zone. We now discuss these results in light of the original hypotheses of spatially variable genetic structuring proposed by Overeem et al. (2008), and new hypotheses resulting from this study, with relevant results summarized in Table 3.

**Table 3.** Hypotheses for spatially variable genetic structuring among colonies of *E.minor* in Australia, and relevant evidence

Hypotheses for spatially variable genetic structuring	Relevant results
Existing hypotheses (Overeem et al. 2008)	
1. Differences in breeding phenology	Genetic homogeneity where phenology differs, and heterogeneity where it is probably similar (Rejects hypothesis)
2. Oceanographic breaks	Genetic breaks between proximate colonies within the same oceanographic zone (within Leeuwin Current), and lack of genetic breaks between some oceanographic systems (Leeuwin vs. East Australian Current) (Rejects hypothesis)
3. Legacy of recent colonization of Bass Strait	Similar IBD relationships in southeastern and western Australia (Rejects hypothesis)
New hypotheses (this study)	
1. Secondary contact of 2 distinct lineages, neutral introgression	Significant IBD relationships in the western, central, and eastern parts of the study range, but the relationship was much steeper in the center. Bayesian clustering under *K* = 2 indicates genetic cline in coancestry coefficients
2. Secondary contact of 2 distinct lineages, selection against interbreeding (i.e., a “Tension Zone”)	Significant IBD relationships in the western, central, and eastern parts of the study range, but the relationship was much steeper in the center. Bayesian clustering under *K* = 2 indicates genetic cline in coancestry coefficients. Inability to reject coincidence of single-locus cline centers
3. Founding or bottlenecking of the Australian lineage in the central zone, and subsequent expansion	Phylogeographic evidence for recent colonization of Australia from New Zealand (Banks et al. 2002; Peucker et al. 2009). Shallower IBD relationships at the peripheries of the Australian range. Lack of difference in signatures of genetic variation across the Australian range (Figure 3)

Overeem et al. (2008) raised 3 hypotheses for contrasting levels of spatial population genetic variation observed within their study range (Table 3): spatial variation in breeding phenology, the role of an oceanographic barrier, or recent establishment of Bass Strait colonies from a genetically homogenous source following marine transgression ~13 000 years ago. Hypotheses of these types have been invoked to explain population genetic structuring of seabirds elsewhere (Friesen et al. 2007a). While we cannot reject these factors as contributors for the genetic structuring observed by Overeem et al. (2008), there is certainly no evidence for their importance based on genetic structuring elsewhere in the Australian range. For instance, significant genetic differences exist between proximate colonies likely to exhibit similar phenology (e.g., Penneshaw and Kingscote, 27 km apart), but are absent between regions with very different phenology (e.g., Phillip Island and Lion Island, 1020 km apart; Rogers et al. 1995). Spatial genetic structuring is also observed among the majority of western colonies despite the lack of intervening oceanographic breaks (all within the Leeuwin Current), and is absent for some comparisons against Lion Island that involve different oceanographic systems (East Australian Current vs. Leeuwin Current). Finally, IBD relationships east of Granite Island appear similar to those west of Troubridge Island, and therefore there does not seem to be any particular significance attributable to the presence of an expansive and recently inundated continental shelf (i.e., Bass Strait) for weaker spatial genetic structuring.

The presence of finer spatial structuring of genetic variation in the Troubridge–Granite Island region and different IBD relationships among regions can be more readily explained by nonequilibrium population genetic structuring (Hutchison and Templeton 1999; Bradbury and Bentzen 2007; Petrou et al. 2014). There are 3 possible causes that each relate specifically to the Troubridge–Granite Island zone (Table 3). There may be secondary contact of cryptic lineages in this region, with either 1) incomplete neutral introgression, or 2) selection against interbreeding between lineages. Alternatively, 3) the entire Australian population may have recently been founded in this region, and is slowly expanding its range.

The smooth clinal transition in coancestry coefficients among colonies within the Troubridge–Granite Island region is consistent with interbreeding and neutral introgression between genetically distinct lineages from either side of this zone (Endler 1977). Similar inferences have been made for common murres *Uria aalge* (Morris-Pocock et al. 2008) and brown skuas *Catharacta antarctica lonnbergi* (Ritz et al. 2008). Isolation of 2 *E.minor* lineages may have occurred via northward movement in response to climate during the LGM, producing allopatric east and west coast populations, as has been hypothesized for other temperate marine Australian taxa (Burridge 2000; Waters et al. 2004; Fraser et al. 2009). This process is analogous to postglacial movements inferred for many Northern Hemisphere taxa (Hewitt 2000; Hewitt 2004). Alternatively, the ephemeral nature of penguin colonies may have resulted in the formation of a central gap in the southern Australian distribution of the species, which promoted the isolation and divergence of lineages either side. Alternatively, anthropogenic predation may have also caused localized extirpation (Boessenkool et al. 2009). The lack of deep mtDNA divergence between eastern and western populations (Peucker et al. 2009) indicates they would not have been long-isolated before secondary contact, but current models to estimate these times ignore gene flow subsequent to admixture.

As an alternative to neutral introgression, the cline may reflect selection against interbreeding following secondary contact (a Tension Zone; Barton and Hewitt 1985; Miraldo et al. 2013; Smith et al. 2013). While cline analysis failed to reject coincidence of microsatellite and mitochondrial cline centers, this would also be expected under very recent secondary contact and neutral introgression (Endler 1977). Observations of mate choice and fitness of pairs within the Troubridge–Granite Island region with respect to their multilocus coancestry coefficients are required to prove selection against interbreeding.

The last possible explanation involves the founding or bottlenecking of the Australian population within the Troubridge–Granite Island region, and subsequent expansion to the east and west. Phylogeographic evidence suggests that the Australian population colonized from New Zealand ~2.5 Myr ago (Banks et al. 2002), and the lack of phylogeographic structuring within Australia (Peucker et al. 2009) is also consistent with a recent expansion. Likewise, the shallower IBD slopes at the peripheries of the Australian range relative to the Troubridge–Granite Island zone are compatible with recent expansion (Castric and Bernatchez 2003). The lack of clear peaks or troughs in indices of genetic variation in the Troubridge–Granite Island zone relative to other parts of the study range (Figure 3) also provide support for this hypothesis over those involving secondary contact of genetically distinct lineages.

While nonequilibrium methods for estimating gene flow have previously been used to address hypotheses such as those raised here (Morris-Pocock et al. 2008), when trialled on our dataset they were either inappropriate with respect to assumptions regarding levels of gene flow (Faubet et al. 2007), or failed to converge (Faubet and Gaggiotti 2008). Likewise, tests of population founder effect can exhibit substantial Type II error in a metapopulation context (Reynolds and Fitzpatrick 2013). Simulation approaches may therefore be more successful at testing these new hypotheses (Petrou et al. 2013). As the populations of *E.minor* in Australia as a whole may not be at migration-drift equilibrium (owing to the regional nonequilibrium scenarios suggested above), traditional interpretations of the genetic structuring observed herein should be avoided (Whitlock and McCauley 1999). Indeed, it is possible that gene flow per unit distance does not vary across the Australian range of this species. Gene flow is unlikely to be large relative to the Australian range of the species, as this would preclude the observation of regionally different IBD relationships, unless colonization or secondary contact was especially recent (Bradbury and Bentzen 2007).

In this study, the spatial distribution of sample sites along a relatively linear coastline aided the recognition of nonequilibrium population genetic structuring. In particular, we sampled at higher density in the area where significant population genetic structuring was observed. In contrast, coarser sampling would likely have led to alternative interpretations; for example, a hard barrier to gene flow would have been invoked in the Troubridge–Granite Island zone if multiple populations were not sampled across this region. Similarly, while deviation from migration–drift equilibrium is commonly considered when population genetic structuring is lower than expected (Friesen et al. 2007a), it is less frequently considered when genetic structuring is higher than anticipated (but see Morris-Pocock et al. 2008). The presence of a tension zone or recent secondary contact and neutral introgression will produce greater population genetic structuring than would be expected under migration–drift equilibrium. Overall, studies should consider nonequilibrium explanations for observed population genetic structuring in the context of their sampling design, and modify it accordingly, even retrospectively (Schwartz and McKelvey 2009; Anderson et al. 2010).

## Supplementary Material

Supplementary material can be found at http://www.jhered.oxfordjournals.org/.

## Funding

Australian Research Council (LP0453481); Phillip Island Nature Park.

## Supplementary Material

Supplementary Data
