# Supplementary material for: Nonequilibrium Conditions Explain Spatial Variability in Genetic Structuring of Little Penguin (Eudyptula minor)
Source: J Hered. 2015 Apr 1;106(3):228–37. doi: 10.1093/jhered/esv009 (PMC4406270; doi:10.1093/jhered/esv009)
Supplement: Supplementary Data [file supp_106_3_228__index.html]

Nonequilibrium Conditions Explain Spatial Variability in Genetic Structuring of Little Penguin (Eudyptula minor) — Nonequilibrium Conditions Explain Spatial Variability in Genetic Structuring of Little Penguin (Eudyptula minor) — Nonequilibrium Conditions Explain Spatial Variability in Genetic Structuring of Little Penguin (Eudyptula minor) — Supplementary Data 

# Nonequilibrium Conditions Explain Spatial Variability in Genetic Structuring of Little Penguin (*Eudyptula minor*)

## Supplementary Data

Data files

**Files in this Data Supplement:**

- Supplementary Data - Supplementary Data
