# Supplementary material for: Nonequilibrium Conditions Explain Spatial Variability in Genetic Structuring of Little Penguin (Eudyptula minor)
Source: J Hered. 2015 Apr 1;106(3):228–37. doi: 10.1093/jhered/esv009 (PMC4406270; doi:10.1093/jhered/esv009)
Supplement: Supplementary Data [file supp_esv009_Burridge_SupMat_all_materials.docx]

Supplementary Material Table 1 Exact test probabilities of homogenous allele frequencies between pairs of colonies.

|  | Penguin Island | Cheyne Island | Pearson Island | Spencer Gulf | Troubridge Island | Kingscote | Penneshaw | West Island | Granite Island | Middle Island | London Bridge | Phillip Island | Gabo Island | Lillico Beach | Bruny Island | Lion Island | Cabbage Tree Island |
| --- | --- | --- | --- | --- | --- | --- | --- | --- | --- | --- | --- | --- | --- | --- | --- | --- | --- |
| Penguin Island |  | <0.001^1^ | <0.001^1^ | 0.001^1^ | <0.001^1^ | <0.001^1^ | <0.001^1^ | 0.001 | <0.001^1^ | 0.397 | 0.003^1^ | 0.003^1^ | 0.122 | 0.059 | 0.129 | 0.260 | 0.008^1^ |
| Cheyne Island | <0.001^1^ |  | 0.013 | 0.071 | <0.001^1^ | <0.001^1^ | 0.022 | <0.001^1^ | 0.009^1^ | <0.001^1^ | <0.001^1^ | <0.001^1^ | <0.001^1^ | <0.001^1^ | <0.001^1^ | <0.001^1^ | <0.001^1^ |
| Pearson Island | <0.001^1^ | <0.001^1^ |  | 0.087 | <0.001^1^ | <0.001^1^ | 0.010 | <0.001^1^ | 0.010 | <0.001^1^ | <0.001^1^ | <0.001^1^ | <0.001^1^ | <0.001^1^ | <0.001^1^ | 0.001^1^ | <0.001^1^ |
| Spencer Gulf | <0.001^1^ | <0.001^1^ | 0.006^1^ |  | <0.001^1^ | 0.373 | 0.336 | 0.021 | 0.044 | <0.001^1^ | <0.001^1^ | <0.001^1^ | <0.001^1^ | <0.001^1^ | <0.001^1^ | 0.022 | <0.001^1^ |
| Troubridge Island | <0.001^1^ | <0.001^1^ | <0.001^1^ | 0.075 |  | <0.001^1^ | <0.001^1^ | <0.001^1^ | <0.001^1^ | <0.001^1^ | <0.001^1^ | <0.001^1^ | <0.001^1^ | <0.001^1^ | <0.001^1^ | <0.001^1^ | <0.001^1^ |
| Kingscote | <0.001^1^ | <0.001^1^ | <0.001^1^ | 0.015 | <0.001^1^ |  | 0.044 | <0.001^1^ | <0.001^1^ | <0.001^1^ | <0.001^1^ | <0.001^1^ | <0.001^1^ | <0.001^1^ | <0.001^1^ | <0.001^1^ | <0.001^1^ |
| Penneshaw | <0.001^1^ | <0.001^1^ | <0.001^1^ | 0.007^1^ | <0.001^1^ | <0.001^1^ |  | 0.001^1^ | 0.007^1^ | <0.001^1^ | <0.001^1^ | <0.001^1^ | <0.001^1^ | <0.001^1^ | <0.001^1^ | <0.001^1^ | <0.001^1^ |
| West Island | <0.001^1^ | <0.001^1^ | <0.001^1^ | 0.001^1^ | <0.001^1^ | <0.001^1^ | <0.001^1^ |  | 0.012 | 0.004^1^ | <0.001^1^ | 0.007^1^ | 0.004^1^ | 0.001^1^ | 0.023 | 0.090 | <0.001^1^ |
| Granite Island | <0.001^1^ | <0.001^1^ | <0.001^1^ | 0.004^1^ | <0.001^1^ | <0.001^1^ | <0.001^1^ | 0.414 |  | <0.001^1^ | <0.001^1^ | <0.001^1^ | <0.001^1^ | 0.001^1^ | 0.002^1^ | 0.046 | <0.001^1^ |
| Middle Island | <0.001^1^ | <0.001^1^ | <0.001^1^ | <0.001^1^ | <0.001^1^ | <0.001^1^ | <0.001^1^ | 0.005^1^ | 0.023 |  | 0.039 | 0.015 | 0.906 | 0.511 | 0.392 | 0.371 | 0.025 |
| London Bridge | <0.001^1^ | <0.001^1^ | <0.001^1^ | <0.001^1^ | <0.001^1^ | <0.001^1^ | <0.001^1^ | <0.001^1^ | <0.001^1^ | 0.616 |  | 0.367 | 0.058 | 0.425 | 0.063 | 0.033 | <0.001^1^ |
| Phillip Island | <0.001^1^ | <0.001^1^ | <0.001^1^ | 0.002^1^ | <0.001^1^ | <0.001^1^ | <0.001^1^ | 0.001^1^ | <0.001^1^ | 0.148 | 0.222 |  | 0.068 | 0.089 | 0.025 | 0.143 | 0.005^1^ |
| Gabo Island | <0.001^1^ | <0.001^1^ | <0.001^1^ | <0.001^1^ | <0.001^1^ | <0.001^1^ | <0.001^1^ | <0.001^1^ | <0.001^1^ | 0.015 | 0.165 | 0.002 |  | 0.632 | 0.346 | 0.274 | 0.082 |
| Lillico Beach | <0.001^1^ | <0.001^1^ | <0.001^1^ | <0.001^1^ | <0.001^1^ | <0.001^1^ | <0.001^1^ | 0.004^1^ | 0.005^1^ | 0.681 | 0.109 | 0.042 | 0.418 |  | 0.432 | 0.376 | <0.001^1^ |
| Bruny Island | <0.001^1^ | <0.001^1^ | <0.001^1^ | 0.001^1^ | <0.001^1^ | <0.001^1^ | <0.001^1^ | 0.014 | 0.152 | 0.765 | 0.733 | 0.725 | 0.901 | 0.989 |  | 0.502 | 0.009^1^ |
| Lion Island | <0.001^1^ | <0.001^1^ | <0.001^1^ | 0.001^1^ | <0.001^1^ | <0.001^1^ | <0.001^1^ | 0.007^1^ | 0.018 | <0.001^1^ | <0.001^1^ | 0.006^1^ | 0.017 | 0.017 | 0.023 |  | 0.039 |
| Cabbage Tree Island | <0.001^1^ | <0.001^1^ | <0.001^1^ | <0.001^1^ | <0.001^1^ | <0.001^1^ | <0.001^1^ | <0.001^1^ | <0.001^1^ | <0.001^1^ | <0.001^1^ | <0.001^1^ | 0.001^1^ | <0.001^1^ | 0.012 | <0.001^1^ |  |

Microsatellite loci below the diagonal, mitochondrial DNA above the diagonal.^1^Significant following Benjamini-Yekutieli correction.

**Supplementary Material Figure 1**Delta *K*and mean LnP(*K*) as a function of *K* (number of inferred clusters) during STRUCTURE analysis. Error bars represent standard deviation.

**Supplementary Material Figure 2** Estimated individual coancestry proportions from STRUCTURE analysis, assuming 2–6 clusters. Each individual is represented by a vertical bar with colours proportional to its estimated coancestry to each cluster. The source of individuals is demarcated along the bottom of the chart.

**Supplementary Figure 3** Cline in first axis scores from multiple correspondence analysis of mitochondrial DNA haplotype frequencies, along a transect spanning southern Australian colonies of *Eudyptula minor* (from Cheyne Island to Gabo Island). The fill of each individual data point was 97% transparent, such that final shading provides an indication of the frequencies of haplotypes with a given score within each colony. Line represents the maximum likelihoodfit of a sigmoidal curve.


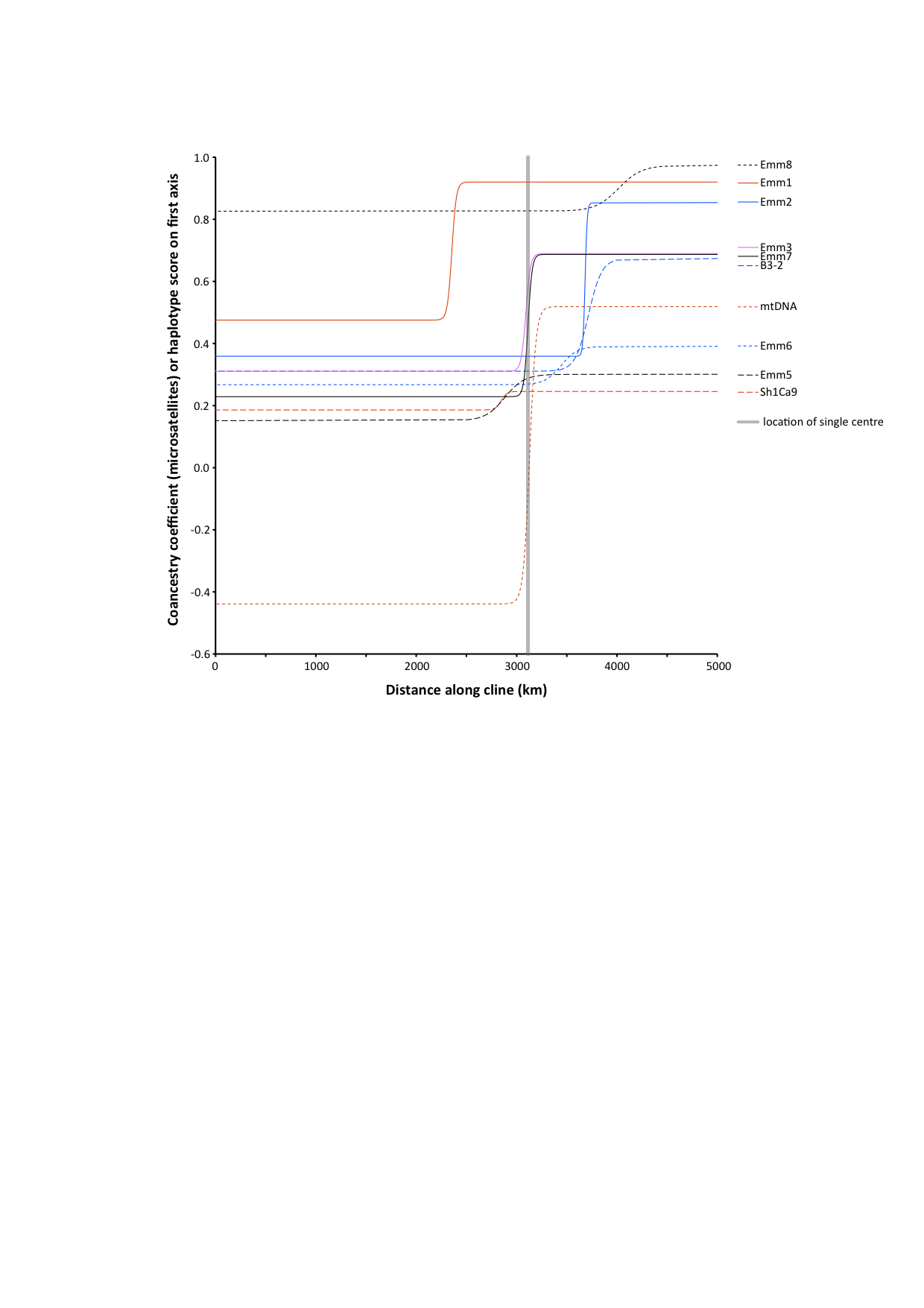


**Supplementary Figure 4** Cline in coancestry coefficients from STRUCTURE analysis of microsatellite loci analysed individually, and first axis scores from multiple correspondence analysis of mitochondrial DNA haplotype frequencies, along a transect spanning southern Australian colonies of *Eudyptula minor* (from Cheyne Island to Gabo Island). The grey vertical line indicates the position of cline centre when all loci are constrained to the same location.
